# Epidemic and Endemic Malaria Transmission Related to Fish Farming Ponds in the Amazon Frontier

**DOI:** 10.1371/journal.pone.0137521

**Published:** 2015-09-11

**Authors:** Izabel Cristina dos Reis, Nildimar Alves Honório, Fábio Saito Monteiro de Barros, Christovam Barcellos, Uriel Kitron, Daniel Cardoso Portela Camara, Glaucio Rocha Pereira, Erlei Cassiano Keppeler, Mônica da Silva-Nunes, Cláudia Torres Codeço

**Affiliations:** 1 Programa de Computação Científica, Fiocruz, Rio de Janeiro, RJ, Brasil; 2 Laboratório Transmissores de Hematozoários, Instituto Oswaldo Cruz, Fiocurz, Rio de Janeiro, RJ, Brazil; 3 Departamento de Zoologia, Universidade Federal de Pernambuco, Recife, PE, Brazil; 4 Laboratório de Informação em Saúde, Instituto de Comunicação e Informação Científica e Tecnológica em Saúde (ICICT), Fiocruz, Rio de Janeiro, RJ, Brasil; 5 Department of Environmental Studies, Emory University, Atlanta, Georgia, United States of America; 6 Universidade Federal do Acre—Universidade da Floresta, Centro Multidiscipinar do Campus Floresta, Cruzeiro do Sul, Acre; 7 Núcleo Operacional Sentinela de Mosquitos Vetores—DIRAC/IOC/VPAAPS, Fiocruz, Rio de Janeiro, RJ, Brasil; 8 Centro de Ciências da Saúde e do Desporto—Universidade Federal do Acre, Rio Branco, Acre; University of Queensland & CSIRO Biosecurity Flagship, AUSTRALIA

## Abstract

Fish farming in the Amazon has been stimulated as a solution to increase economic development. However, poorly managed fish ponds have been sometimes associated with the presence of *Anopheles* spp. and consequently, with malaria transmission. In this study, we analyzed the spatial and temporal dynamics of malaria in the state of Acre (and more closely within a single county) to investigate the potential links between aquaculture and malaria transmission in this region. At the state level, we classified the 22 counties into three malaria endemicity patterns, based on the correlation between notification time series. Furthermore, the study period (2003–2013) was divided into two phases (epidemic and post-epidemic). Higher fish pond construction coincided both spatially and temporally with increased rate of malaria notification. Within one malaria endemic county, we investigated the relationship between the geolocation of malaria cases (2011–2012) and their distance to fish ponds. Entomological surveys carried out in these ponds provided measurements of anopheline abundance that were significantly associated with the abundance of malaria cases within 100 m of the ponds (*P* < 0.005; r = 0.39). These results taken together suggest that fish farming contributes to the maintenance of high transmission levels of malaria in this region.

## Introduction

Malaria has reemerged in the Amazon region following the introduction of large-scale agricultural colonization projects [1, 2]. Since the 1970’s, the annual incidence has increased more than tenfold in this region, which has been attributed to the large inflow of migrants to the Amazon region [3, 4]. The reemergence of malaria in this setting has been referred to as “frontier malaria” [5].

An average of 310,390 malaria cases have been reported annually in the Brazilian Amazon during the last 14 years, but with a clear reduction in the last 4 years. Between 2000 and 2014, the number of reported cases dropped from 615,247 to 138,338, despite the population growth [6]. In Brazil, practically all (99.9%) malaria cases are concentrated in the Amazon region, and within this region, distribution is highly focal: only 37 counties, out of 808, reported 80% of all cases in 2013 [2]. Four of these counties are located in the State of Acre: Cruzeiro do Sul, Mâncio Lima, Rodrigues Alves and Tarauacá.

In recent years, as a measure to reduce deforestation and improve the local economy, fish farming, mostly performed in already deforested and abandoned areas, near or within small dammed rivers was stimulated as a sustainable alternative to cattle-farming. However, there has been a growing concern that fish ponds may increase malaria incidence, since *Anopheles darlingi*, the most important Neotropical malaria vector, seems to have adapted well to fish ponds, despite predation by fish juveniles [7–9]. This species has a natural preference for deep, stable, crystal clear water collections, often in proximity to human dwellings. In the Iquitos-Nauta road, Peru, Simpson et al.[10] found higher numbers of self-reported malaria episodes in households that were located closer to fish ponds, which were the most commonly positive larval habitats in this area. In the same area, Maheu-Giroux et al. [11] found evidence of fish pond density as a major risk factor for malaria transmission. In Cruzeiro do Sul, Acre, Costa et al. [12] observed an increased malaria transmission after the excavation of 179 new fish ponds in 2005.

In 2005–2006 a large malaria epidemic struck Acre, with over 80,000 cases reported in two years. In subsequent years, the number of cases was reduced to a new baseline, higher than previously observed [13]. In this study, we analyze malaria transmission during one decade in the State of Acre, from 2003 to 2013, with a special focus on one of the epidemic-stricken counties, Mâncio Lima, where spatial analysis was performed combining detailed epidemiological, entomological and environmental data to investigate the relationship between malaria transmission and fish farming.

## Materials and Methods

### Study area

**Fig 1:**
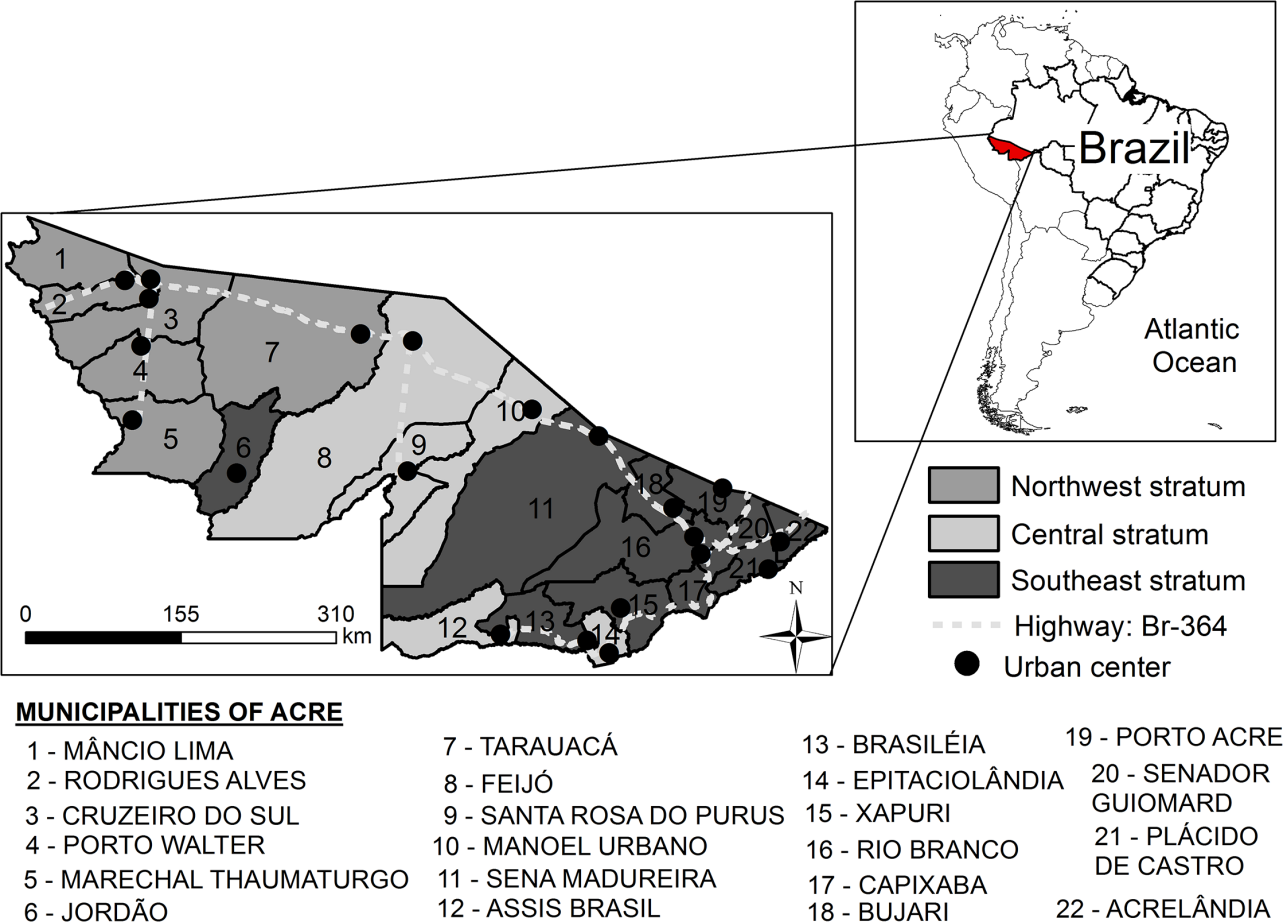
Location of the counties of Acre state, Brazil.

The study was conducted at two scales: the state of Acre (regional) and the county of Mâncio Lima (local). The Brazilian state of Acre, bordering Bolivia (Southeast), Peru (West and North) and the Brazilian States of Amazonas (North) and Rondônia (East) (Fig 1), is divided into 22 counties. The population of Acre in 2014 was 776,463 inhabitants most of whom live in rural areas. Open rain forest with palm and bamboo comprise the main vegetation type [14]. The climate is tropical with annual precipitation ranging from 1,600 to 2,750 mm, highest during November—April. Mean temperature is 24.5°C—32°C, and humidity is always high (> 90%) [15].

Mâncio Lima (4,672 km^2^) (Fig 1), the westernmost county of Acre, has 67 percent of its territory occupied by the Nukini and Poyanawa Indigenous Reserves and a fraction of the Serra do Divisor National Park. The rural economy is based on agriculture and manioc flour production, and no area is licensed for mining exploration [16]. The county town has 8,750 inhabitants and is located along the Japiim river, in an igapo forest, crisscrossed by small streams and wetlands covered with palm trees (*Mauritia flexuosa)*. This environment is favorable for the development of aquaculture, and Mâncio Lima is one of the main targets of fish pond construction in the state.

### Epidemiological data

Malaria notifications per county from 2003 to 2013 were obtained from the Brazilian Official Malaria Notification System (SIVEP-Malaria). In Acre, Community health agents regularly perform thick blood smears to verify the presence of malaria parasite species in residents presenting non-specific acute febrile symptoms and their household contacts. Results were not confirmed with Polymerase Chain Reaction (PCR). The Annual (and Monthly) Parasite Index (API and MPI, respectively) were calculated as the number of positive malaria exams per year per 1,000 inhabitants. An index of malaria vulnerability, Rc, was calculated as the ratio between the number of autochthonous cases and the number of imported cases per year. R_c_ <1 indicates poor conditions for local transmission[17].

For the analysis, we split the study period into two parts (2003–2006 and 2007–2013), representing the epidemic and post- epidemic phases, since changes were anticipated following the major control initiatives implemented from 2006 onwards. In 2006, at the peak of epidemic, most cases were identified by active surveillance (65%), and 71% patients received treatment within 48 hours of symptom onset [18]. Malaria cases reported in 2011 and 2012 in the Mâncio Lima town were georeferenced with Global Positioning System (GPS) devices and imported into ArcView 10.1software.

### Hydrology and Entomological data

With the aid of satellite imagery from OpenStreetMap (October 12, 2011), an open source product that provides free world-wide geographic datasets [19], of the study area and inquiries with the local population, we mapped all fish ponds within the urban area of Mâncio Lima, in 2011. A sample of 55 fish ponds and 38 other water collections (stream-flooded wetlands, creeks, puddles and riverine areas) were monitored biannually in February 2012, July 2012, February 2013 and July 2013 using a standard 0.5-liter dipper (Bioquip Co., Gardena, CA, USA) as previously described [20]. Since it has been previously noted that *Anopheles* (*Nyssorhynchus*) larvae are distributed in the margins of water collections and are rarely found away from them [21–23] and consequently only the perimeters of the aquatic habitats were sampled. Since data on the size and number of ponds were not available, hectares of aquaculture in Mâncio Lima were estimated based on fish yield as calculated in the neighboring state of Amazônia (1_hectare of aquaculture constructed per year = 70 kg of fish) [24]. We used the reported fish harvest production data [25] and this transformation factor to estimate the number of hectares of aquaculture constructed per year, from 2003 to 2006. In other counties of Acre, the number of fish farms built per year, from 2011 to 2014 was obtained from SEAPROF (State Department of Agroforestry Extension Family). In some counties, where data were missing, an estimate was obtained from Google Earth and local reports. Multispectral band images with 30 m/pixel spatial resolutions where obtained for various dates, between 2003 and 2014 from Landsat 7TM, NASA Landsat Program (Washington, D.C.). High resolution (6 m) multispectral images were also obtained from the SPOT 6TM satellite (Toulouse, France). Images are currently available in the public domain and may be viewed through Google EarthTM (http://www.google.com/earth/).

### Statistical analysis

To visualize the spatial and temporal distribution of malaria incidence in Acre, maps and time series plots were created using GIS (ArcView). To aggregate counties in strata, we applied a hierarchical clustering technique to the monthly time series of MPI per county. To partition the counties into clusters, we used the package ClustOfVar [26] in software R 3.1.0 [27] where a cluster of counties is defined by the strength of the link of each time series to a central synthetic variable, as measured by the squared Pearson correlation. The adequacy of the grouping is measured in terms of proportion of cohesion (%). To compare malaria incidence and fish pond construction between strata, we used ANOVA and Wilcoxon’s matched pair tests.

In Mâncio Lima, where primary data was collected, a map with the location of all malaria cases in 2011 and 2012, as well as the fish ponds, was created. Buffers 100 to 1000m around fish ponds were constructed and the number of malaria cases per buffer, calculated. An exponential regression model following the expression *(Number of malaria cases) = b0 exp (b1xdistance)* was fitted to the data using the least squares technique. Statistical analyzes were performed using StatSoft Inc. (2011). Data is available in S1 File.

### Ethical approval

The study was approved by the ethical committee (CEP Fiocruz n.402.039). Entomological collections were carried out in private and public spaces. Access to private spaces was requested to each land owner, and collections carried out only after their oral consent. No personal information was recorded or used in this study. Access to public spaces did not require permission but before taking place, the overall study was presented and approved by local Health and Environmental Secretariats. The only request was that the results were presented to the population, which occurred in the form of talks in the city's conference room and in schools. The field studies did not involve endangered or protected species.

## Results

**Fig 2:**
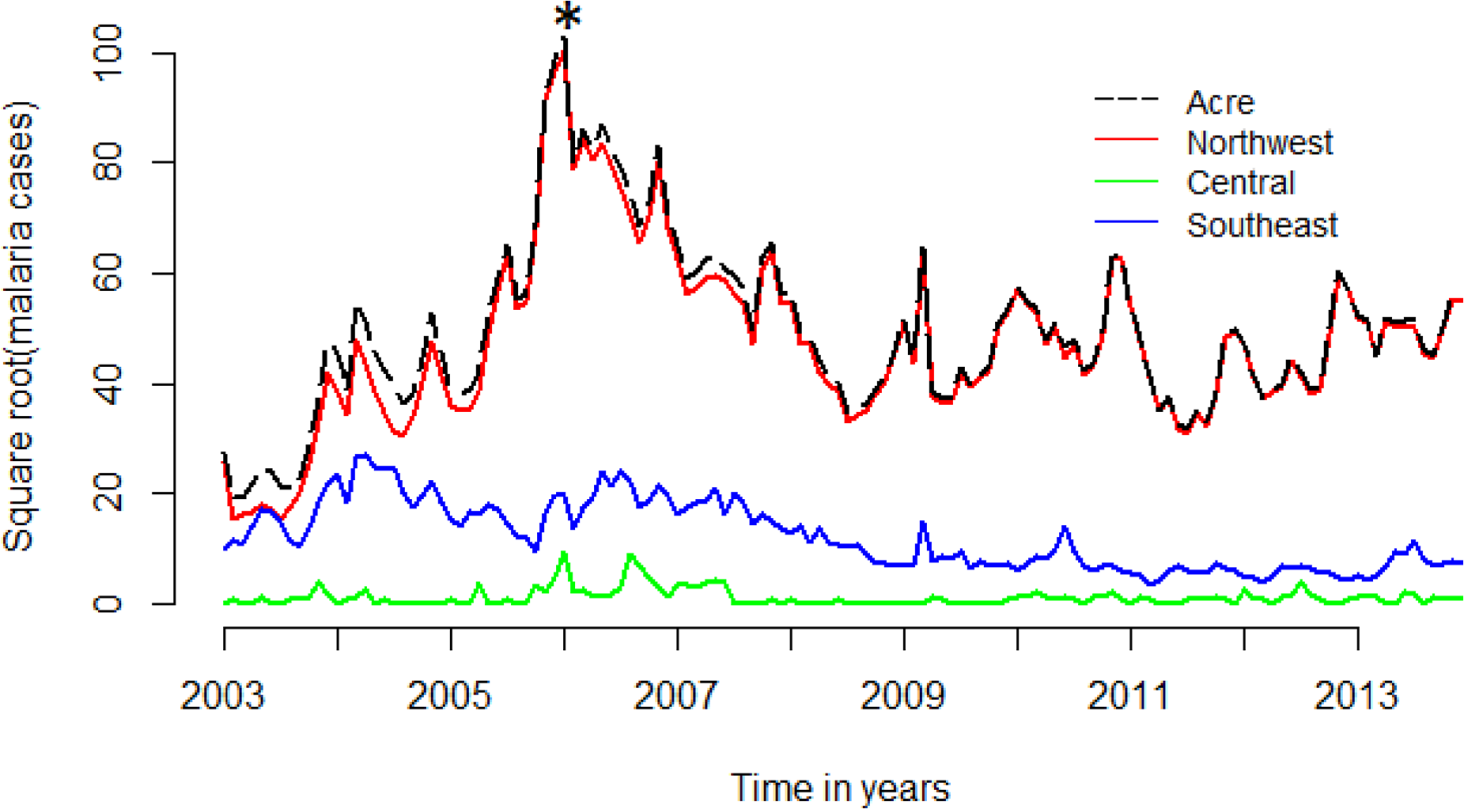
Time series of malaria autochthonous cases in Acre and per stratum, 2003–2013. (*) marks the 2006 epidemics when control actions were strengthened in this region.

From 2003 to 2013, 401,312 malaria cases were notified in Acre, 94% of which were classified as autochthonous. The majority of autochthonous cases (79%) occurred in the rural areas. Acre malaria cases from 2003–2013 are shown in Fig 2.

Correlations between times series of reported autochthonous malaria cases per county from 2003 to 2013 are shown in the form of a dendogram (Fig 2). A strong correlation was observed among all six counties located in Northwest Acre: Cruzeiro do Sul, Mâncio Lima, Marechal Thaumaturgo, Porto Walter, Rodrigues Alves and Tarauacá. These counties are connected by a paved road and/or waterways and a large number of individuals and commodities move between them. They form a group referred to as the Northwest stratum. At the other geographical extreme of the state, the Southeast, 11 neighboring counties also showed consistent correlations among their malaria times series, although less intense than the Northwest stratum. They share malaria dynamics characterized by intense transmission during the early 2000's, followed by a steady decrease. They are Acrelândia, Brasiléia, Bujari, Capixaba, Jordão, Plácido de Castro, Porto Acre, Rio Branco, Sena Madureira, Senador Guiomard, Xapuri. These 11 counties, aggregated into the Southeast stratum, are characterized by a greater connection to other countries and states, larger urban populations and larger areas devoted to cattle farming and agriculture.

**Fig 3:**
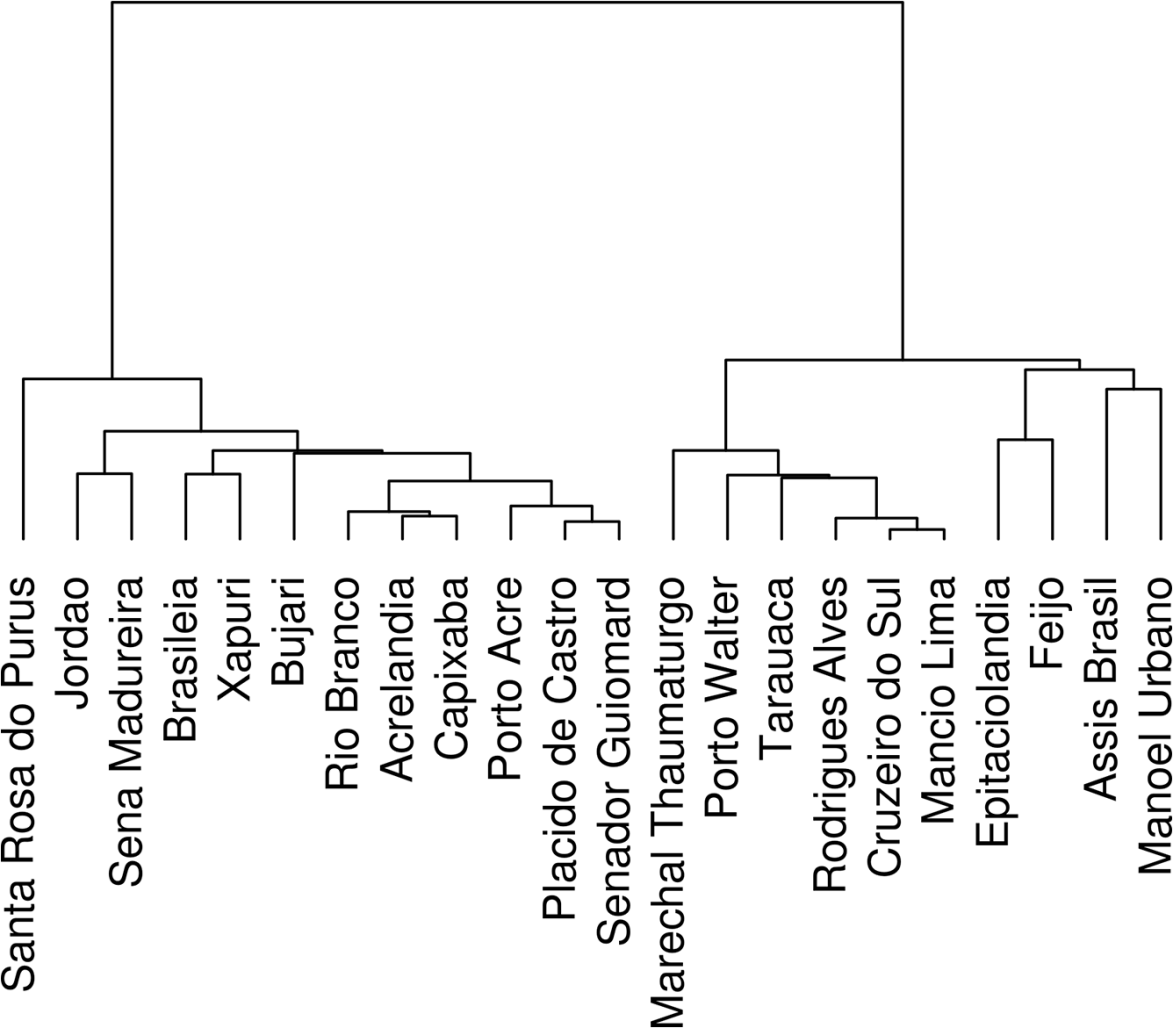
Hierarchical clustering of the 22 municipalities of Acre according to the similarity of the time series of malaria incidence (2003–2013).

In the middle of the state, 5 remaining counties were grouped into the Central stratum. They share low malaria activity, low connectivity to other regions and mostly rural populations. They are Assis Brasil, Manoel Urbano, Feijó, Epitaciolândia and Santa Rosa do Purus. Although this last county is grouped with the Southeast stratum counties in the cluster dendogram, it shows low malaria activity like the Central stratum counties and we opted to assign it to the Central cluster (Fig 3).

Considering the 2003–2013 period as a whole, API presented significant differences between the 3 strata: while the Northwest stratum (n = 6) had a median MPI of 10.8 cases per 1,000 inhabitants (min-max = 0 to 188), the Central stratum (n = 5) had a median of 0 cases/1,000 inhabitants (min-max = 0 to 2.3) and the Southeast stratum (n = 11) 0.44 cases/1,000 inhabitants (min-max = 0–23) per month.

Regarding Rc, the Northwest stratum presented Rc > 1 in 31% of the months from 2003 until 2013, contrasting with the Southeast stratum, with only 12% of months with Rc >1. Transmission was lowest in the Central stratum, with Rc > 1 in only 5% of the months.

**Fig 4:**
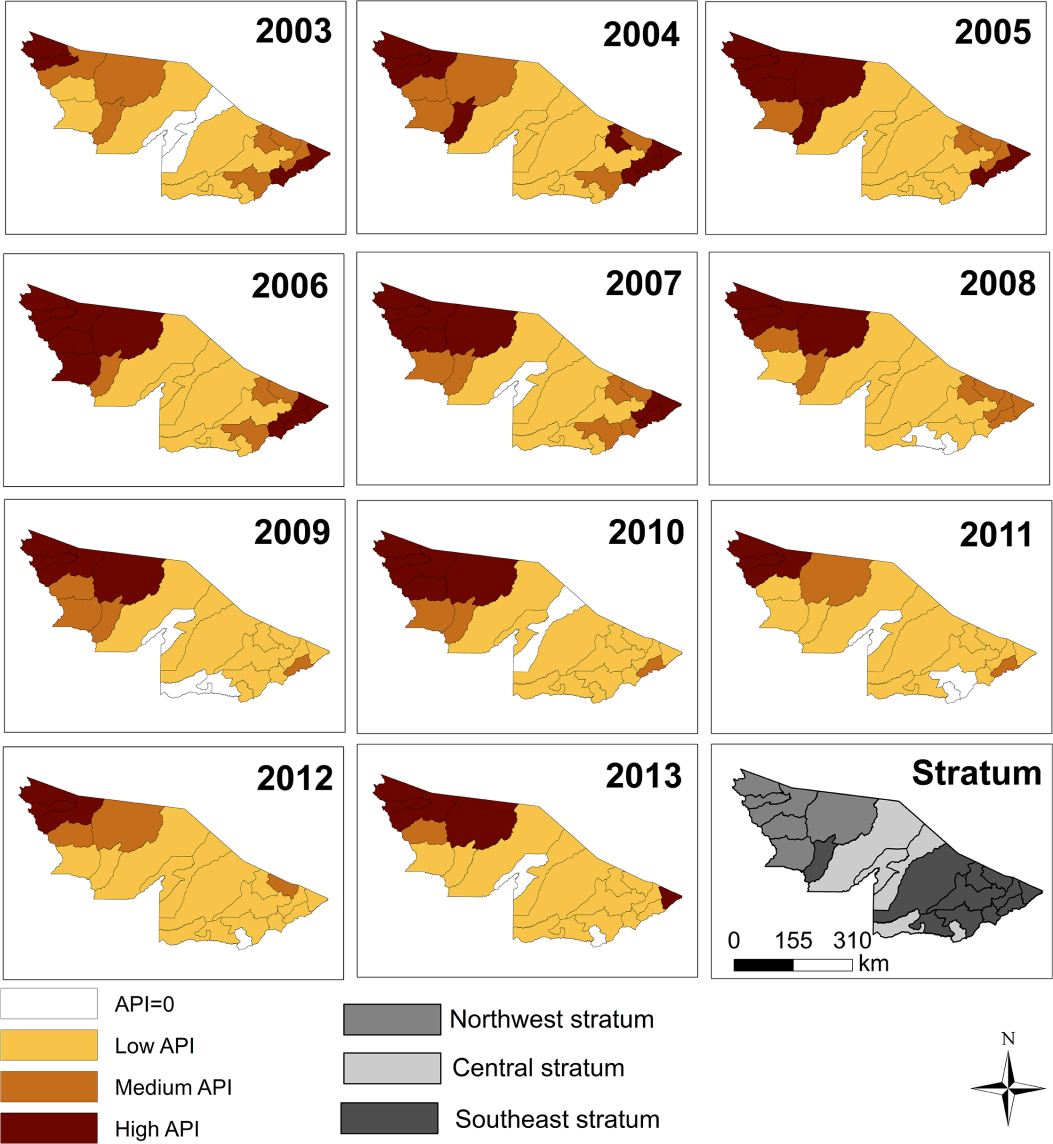
Distribution of Annual Parasitic Index by county in Acre State, from 2003 to 2013. API = 0: No risk; Low API: < 10 cases; medium API: ≥ 10 and < 50 cases; high API: ≥ 50 cases.

A sequence of maps shows how the API varied from 2003 to 2013 in Acre counties (Fig 4). Between 2003 and 2007, two clusters of high API values were seen, one in the Northwest stratum, and one in the Southeast stratum. The Northwest cluster persisted through the whole decade. The Southern cluster, on the other hand, had higher malaria rates until 2007. After 2007, until 2012, the incidence steadily decreased although never becoming null. In 2013, Acrelândia (Southeast stratum), once more presented a high API.

### Malaria and fish farming in Acre

Fish farming initiatives began in Cruzeiro do Sul before 2004, but the greatest number of ponds were built in 2005 [12]. In Mâncio Lima, Rodrigues Alves and Tarauacá, most ponds were built in 2005. The counties with the greatest number of fish ponds were Mâncio Lima, Cruzeiro do Sul, Tarauacá and Rodrigues Alves (mean of 100.1; SD = 12.9). Feijó had lower mean pond construction rates (50.7 ponds/year; SD = 35.7) and fewer ponds than these four counties. The mean construction rate differed significantly between counties by Wilcoxon’s matched pair test (P<0.05).

Counties with the construction rate higher than 80 fish ponds per year during the 2003–2006 study period differed significantly in malaria rates from those with lower construction rates (F(1, 20) = 13.32; *P* < 0.005): while counties with high construction rates (n = 6) were associated with a mean of 833.8 (SE = 177.1) cases of malaria/1000 inhabitants/year, counties with the construction of fewer ponds (n = 16) had a mean malaria incidence of only 76.2 cases/1000 inhabitants-year (SE = 76.2).

### Malaria and fish farming in Mâncio Lima

**Fig 5:**
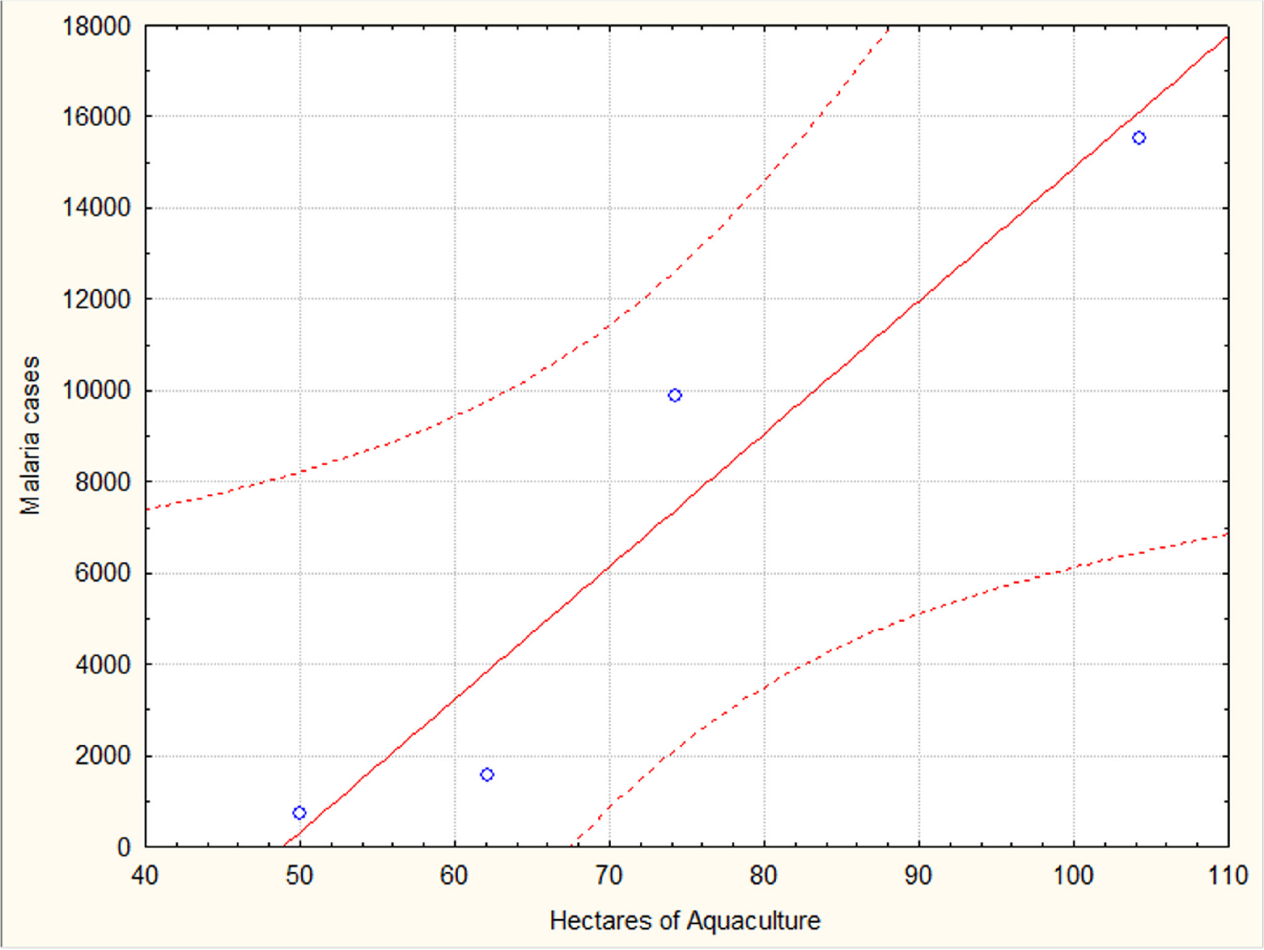
Number of hectares of aquaculture in Mâncio Lima estimated from harvest data in 2003 to 2006, as related to the number of malaria cases in the county.

The number of aquaculture hectares between 2003 and 2006 was directly correlated with malaria incidence in Mâncio Lima through the equation: *y* = -14223 + 290*x (*r* ^2^ = 0.92, *P* < 0.05); where *x* is the number of estimated aquaculture in Mâncio Lima and *y* is the number of malaria cases per year in the county, as shown in Fig 5.

**Fig 6:**
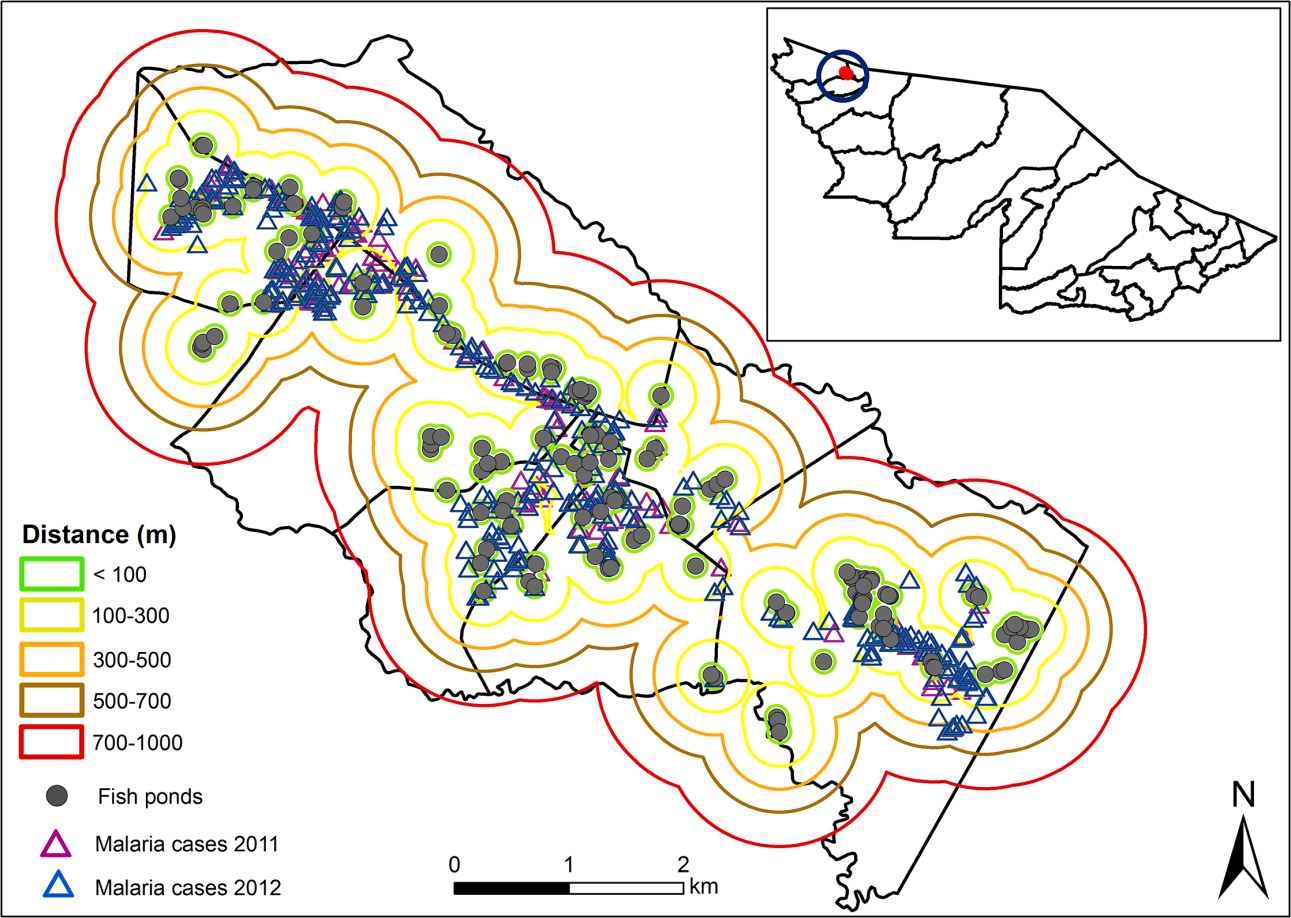
Buffer zone from 100 to 1000m around breeding sites in the urban/periurban area of Mâncio Lima, and malaria cases in 2011 and 2012.

**Fig 7:**
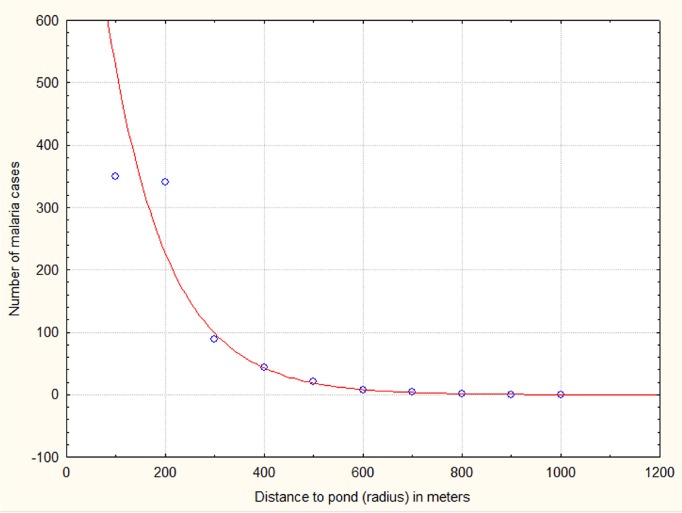
Scatterplot of the number of malaria cases in 2011 and 2012 as related to the distance to the nearest fish ponds, in meters.

A map of fish ponds and malaria cases in the Mâncio Lima town in 2011 and 2012 is shown in Fig 6. Buffer zones around fish ponds are also shown. Fish farming in Mâncio Lima is mostly carried out in small farms or in the backyards. These ponds were constructed by digging or by damming small streams. It is common to find fish ponds close to palm tree rows, where the litter creates refugia for anopheline larvae. The number of malaria cases in 2011 and 2012 was related to the proximity to the nearest fish pond by the equation: Number of malaria cases = 1201.7 exp (-0.008* x) where x is the distance in meters (Fig 7). Malaria cases were absent > 900 m from fish ponds.

### Entomological data

The proportion of fish ponds (n = 55) positive for *An*. *darlingi* larvae was 74.1%, twice as high as the other water collection types (n = 37) (F(4, 90) = 8.24; *P* << 0001). During the four collection periods, a mean of 1.94 times (SE = 0.17), fish ponds were found positive while the second most positive habitat, riverine areas, were positive only a mean of 0.67 times (SE = 0.73). The abundance of *An*. *darlingi* larvae was higher in fish ponds than in any other water collection types (F(4, 90) = 4.37, P < 0.005); whereas the mean larval density in other water collections were at a maximum of 0.67 larvae/dip (SE = 4.60), fish ponds had a mean density of 7.16 (SE = 1.05). The density of *An*. *darlingi* larvae found in each positive fish pond was linearly associated with the number of malaria cases within 100 m from the ponds (*P* < 0.005; r = 0.39). Other anophelines were also found in these ponds but in lower abundance than *An darlingi*. A more detailed description of the chemical and environmental factors associated with anopheline abundance will be presented elsewhere. For the present study, the focus is the association between anopheline abundance and malaria.

## Discussion

Large investments have been made to reduce the burden of malaria in Brazil. Since 2000, large scale distribution of free treatment, supervised treatment administration within 48 hours of symptoms onset, distribution of impregnated bednets, among other strategies have been implemented in Acre, which has received successive prizes by OPAS, suggesting these are winning strategies [28]. However, despite these efforts, malaria remains an important public health problem in the Amazon and, in particular, in Acre. Analyzing malaria epidemiology in this region is important, not only to improve local control strategies, but also to learn about the feasibility of current control goals. Malaria is a focal disease, presenting specific characteristics according to ecological, sanitary, social, political and cultural traits of each locality [29]. This study suggests that the challenges are enormous and differ in each of the three strata of the state of Acre.

Malaria notifications in Acre were highest in the Northwest stratum. At a more regional scale, Braz et al. [30] identifies these counties as belonging to a larger cluster of Amazonian counties with high risk of persistent malaria transmission. The high receptivity to malaria is likely due to the favorable environmental conditions as well as the social conditions that promote close human-vector contact. In common with the Amazonian region identified by Braz et al. [30], the Northwest stratum presents high forest coverage, a superficial waterbed, and larger areas with igapós (periodically flooded forests).

Our results suggest that fish-farming has contributed to the receptivity of the Northwest stratum to malaria. This is supported by the following findings: 1- while contemplating all the counties in Acre from 2003 onwards, the Fish farming Incentive Program concentrated most of its efforts in the Northwest stratum, and higher fish pond construction rates per stratum coincided with higher malaria rates; 2- counties with higher fish pond construction rates were associated with higher mean annual malaria incidences during the epidemic period (2003–2006); 3- the number of hectares of aquaculture in Mâncio Lima was related to malaria incidence in the epidemic period; 4- although we lack fine-scale spatial data during the 2006 epidemic, increased transmission in the post-epidemic period coincides precisely with the occurrence of ponds, since cases decrease exponentially with increasing distance from ponds; 5- fish ponds were the most positive type of *An*. *darlingi* larval habitat, the most constantly positive habitats and the ones with the highest *An*. *darlingi* larval densities; 6- larval densities in fish ponds were associated with the number of malaria cases within 100 m of ponds. Therefore, we believe that fish ponds played a major role in the 2003–2006 epidemics and continued to be an important factor in maintaining higher malaria incidence rates during subsequent years.

A few reports have demonstrated increased malaria transmission in proximity to farming ponds in South America and Africa [10,11,31,32]. In Cruzeiro do Sul, Acre, a recent study has suggested that the location of fish farming ponds coincided with malaria cases in the periphery of the urban center during the 2006 epidemic, although spatial statistics were not reported [12]. In Acre, most fish ponds (73.5%) are created by blocking streams with raised earth barriers [33].

We observed a direct association between malaria prevalence and *An*. *darlingi* larvae abundance in fish ponds at distances of less than 100m. Studies of *An*. *darlingi* flight range in inhabited deforested areas have found that the numbers of mosquitoes flying a given distance from the larval habitat decreases exponentially following the regression equation: y = 4.43*exp(-0.003 x) (P < 0.001, r = 0.98), where x is the distance from the larval habitat in meters and y the percentage of emerging adult *An*. *darlingi* females [34]. Using this equation, it can be determined that 73.4% of emerging females will fly 100 m, but only 21.3% will fly 500 m and 4.6% will fly 1000 m. The percentage of emerging adult females is directly correlated with the number of malaria cases in Mâncio Lima in 2011 and 2012 through the linear regression equation: y = -64.06 + 5.70*x (r = 0.93; P << 0.001; r^2^ = 0.87), where y is the proportion (%) of *An*. *darlingi* adult females and x is the number of malaria cases. These results support the hypothesis that malaria incidence in Mâncio Lima in 2011 and 2012 was closely linked to fish farming ponds.

We hypothesize that fish ponds are important anthropogenic modifications that account for increased malaria transmission. Using health district-specific malaria incidence data from Mâncio Lima, in 2006, higher malaria risk in areas with greater deforestation percentage change in the 1997–2000 period were interpreted by Olson et al.[24] as an indicator that deforestation was a causal factor for malaria. However, the health districts associated with percentage change in the 1997–2000 periods were located along the main axis of the urban center, especially to the south and west, areas where most fish ponds were built. The conclusions presented by Olson et al. [24] were partly based on the results reported by Vittor et al. [22,35], who identified increased *An*. *darlingi* landing catches, as well as larval occurrence, in deforested areas. However, neither of these entomological studies adequately controlled for the presence of fish ponds, although these habitats were recognized as the most frequently positive habitat in the given study area. The association of malaria incidence with proximity to fish ponds and pond density, suggests that the presence of ponds may have biased the entomological data [10,11]. Within the Northwest stratum, the 3 counties with the greatest degree of percent deforestation (Mâncio Lima, Rodrigues Alves and Cruzeiro do Sul) received the greatest incentives for fish pond construction in 2003. Overall, our results confirm that economic activities and land use patterns that take place after deforestation may play an important role in sustaining malaria transmission [36].

## Concluding remarks

Aquaculture is an important driver of development in the Amazon, and has been advocated as a solution to alleviate poverty and foster economic growth. Fish production in Mâncio Lima may have actually improved the local socioeconomic conditions, not only by introducing a new source of income, diversifying food supplies, but also by creating a chain of secondary production and consumption items. S1 Fig shows the variation of Gross Domestic Product (in dollars) per capita from 2000 to 2012 in Mâncio Lima municipality. A sharp increase of GDP per capita can be observed, with an inflexion in 2006, coinciding with fish ponds implementation in Mâncio Lima. Moreover, the contribution of the agricultural sector to GDP has been increasing along this period, from 20 to 30%, possibly as a result of the local fish production. However, human activity may potentially change the pathogenic potential of landscapes, and aquaculture has been demonstrated to be an activity that may impact malaria transmission. Investments in aquaculture should be followed by a specifically designed malaria control program, since risk factors are likely to differ from malaria acquired in less modified environments. More studies are necessary to improve our understanding of the importance of fish ponds, the role of the forest fringe and of how the deforestation process affects *An*. *darlingi* bionomics. The importance of fish ponds’ size, density and bordering vegetation must be better evaluated to derive adequate economic and ecological management of fish ponds and guidelines for malaria surveillance and control. The way different types of secondary growth affect mosquito bionomics is also of great importance and merits further investigation.

## Supporting Information

S1 Fig Variation of GDP per capita (bar) and Proportion of Agriculture (%) (line) from 2000 to 2012 in Mâncio Lima municipality. (TIF)

S1 File Zip file containing the dataset used in this study. It consists of six.csv files, API.csv contains the API each municipalities per year, cluster.csv contains the MPI of each municipalities per year, buffer_cases.csv contains the number of malaria cases per distance zones, entomological.csv contains mean of *An*. *darling* in fish ponds and other water bodies, hectar_aqua.csv contains the hectares of aquaculture data in Mâncio Lima, ponds_constructed contains the ponds constructed per year data per each municipalities.
(RAR)
