# Supplementary figures and images for: Epidemic and Endemic Malaria Transmission Related to Fish Farming Ponds in the Amazon Frontier
Source: PLoS One. 2015 Sep 11;10(9):e0137521. doi: 10.1371/journal.pone.0137521 (PMC4567347; doi:10.1371/journal.pone.0137521)

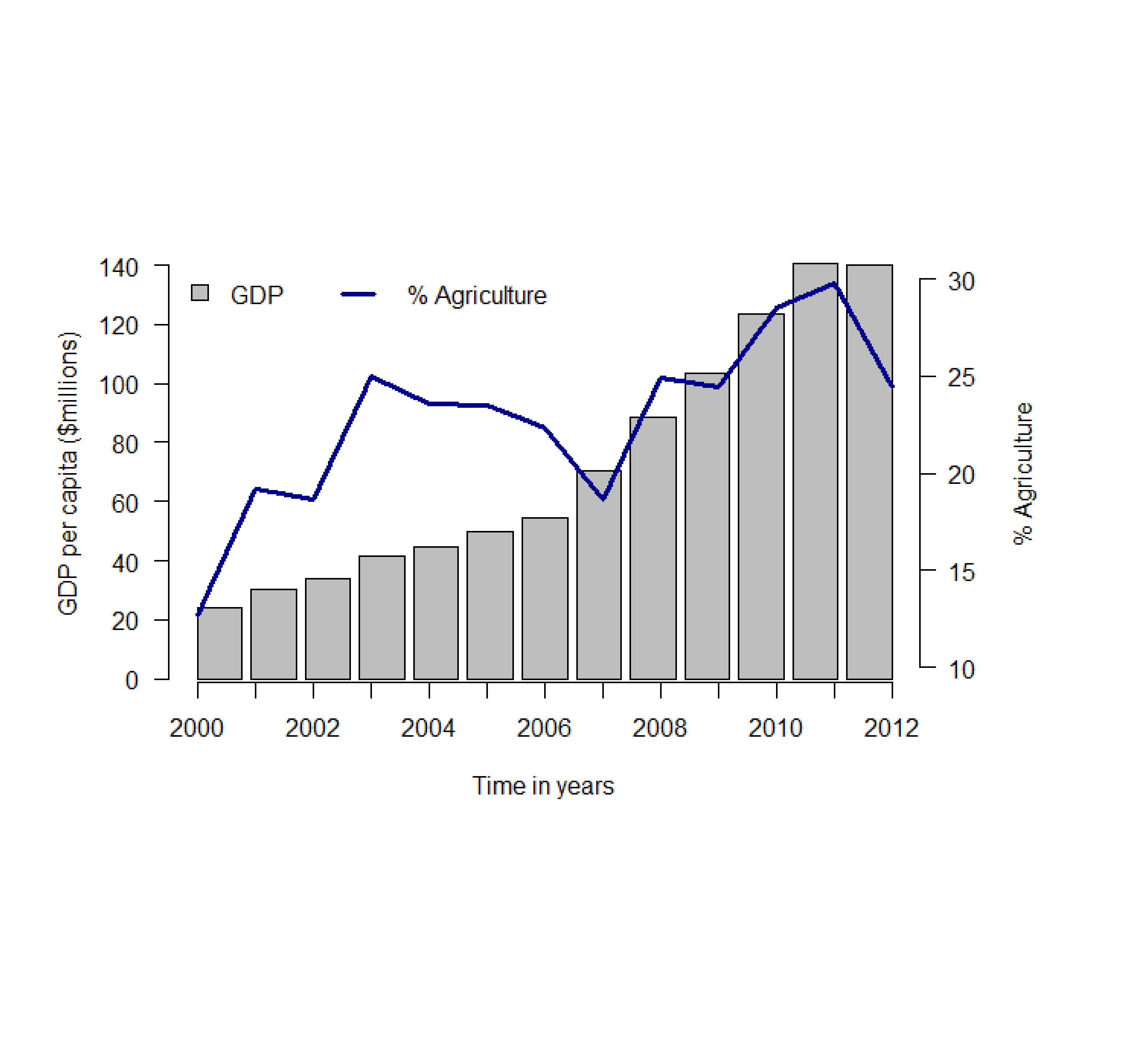

Supplement: S1 Fig — (TIF) [file pone.0137521.s001.tif]
